# Clinical utility of ^18^F-FDG PET/CT imaging in patients with pulmonary artery sarcoma

**DOI:** 10.1186/s13550-022-00890-2

**Published:** 2022-04-04

**Authors:** Jingyun Ren, Huiting Li, Qing Zhang, Entao Liu, Baozhen Zeng, Yan Huang, Lan Wang, Lei Jiang

**Affiliations:** 1 PET Center, Department of Nuclear Medicine, Guangdong Provincial People’s Hospital, Guangdong Academy of Medical Sciences, 106 Zhongshan Er Road, Guangzhou, 510080 China; 2 Department of Cardio-Pulmonary Circulation, Shanghai Pulmonary Hospital, School of Medicine, Tongji University, 507 Zhengmin Road, Shanghai, 200433 China; 3 Department of Pathology, Guangdong Provincial People’s Hospital, Guangdong Academy of Medical Sciences, Guangzhou, China; 4 Department of Pathology, Shanghai Pulmonary Hospital, School of Medicine, Tongji University, Shanghai, China; 5 Guangdong Provincial Key Laboratory of Artificial Intelligence in Medical Image Analysis and Application, Guangzhou, China

**Keywords:** Pulmonary artery sarcoma (PAS), FDG, PET/CT, SUVmax, Overall survival (OS)

## Abstract

**Background:**

Pulmonary artery sarcoma (PAS) is a rare and fatal malignancy. Due to the lack of specific clinical and radiological features, PAS is always misdiagnosed as pulmonary thromboembolism (PTE). This study aimed to investigate ^18^F-FDG PET/CT in distinguishing PAS from PTE, and analyze its correlation with clinical and radiological findings and outcome of PAS.

**Methods:**

Clinical, contrast-enhanced CT, and ^18^F-FDG PET/CT characteristics of 14 patients with PAS and 33 patients with PTE were retrospectively reviewed. The correlation between PET/CT metabolic parameters vs. clinical and CT findings was investigated in patients with PAS. The overall survival (OS) was analyzed in PAS patients.

**Results:**

The SUVmax of PAS (median: 8.0, range 3.0–17.2) was significantly higher than PTE (1.8[0.8–3.7]) (*P* < 0.001), and at a cutoff value of 2.9, the sensitivity and specificity were 100.0% and 93.9%, respectively. Compared with PTE, PAS more frequently occurred in younger population (*P* = 0.011), involved pulmonary trunk (*P* < 0.001), and displayed higher enhanced CT (*P* < 0.001) and ΔCT (enhanced CT compared to non-enhanced CT) (*P* < 0.001) values. SUVmax of PAS was associated with tumor staging (*P* = 0.022) and enhanced CT (*P* = 0.013) and ΔCT (*P* = 0.005) values. The median OS of PAS patients was 10.5 months, and 12-month and 24-month OS rates were 58.0% and 12.0%, respectively. Only D-dimer level (*P* = 0.038) and tumor staging (*P* = 0.019) were associated with OS.

**Conclusions:**

Most PAS displayed high glucometabolism, and SUVmax of ^18^F-FDG PET/CT was useful in distinguishing PAS from PTE.

## Background

Pulmonary artery sarcoma (PAS) is a rare and aggressive malignancy arising from mesenchymal cells of the intima of the pulmonary artery. The clinical and conventional radiological findings of PAS are always similar to pulmonary thromboembolism (PTE) [1–5]. Thus, there is still no consensus regarding the accurate diagnosis of PAS. If a delay in the diagnosis of PAS occurs, the patients will miss the best treatment opportunities, which can lead to inoperable or rapid deterioration conditions. On the contrary, if PTE is misdiagnosed as PAS, the patients will suffer from unnecessary surgery or invasive biopsy.

^18^F-FDG PET/CT imaging provides metabolic and morphologic information of the lesion, which has been established as an effective modality in the differential diagnosis between malignant and benign diseases. However, due to the rare and fatal nature, the studies about ^18^F-FDG PET/CT findings in PAS are quite limited, which has mainly been reported in sporadic cases and small series [5–9]. Hence, the utilization of ^18^F-FDG PET/CT in PAS is still needed to be further investigated. Besides, contrast-enhanced CT is the most common imaging modality for patients with pulmonary artery diseases. Thus, this study retrospectively compared clinical, contrast-enhanced CT, and ^18^F-FDG PET/CT characteristics between the patients with PAS and PTE, evaluated the correlation of ^18^F-FDG PET/CT metabolic parameters with clinical and radiological findings in the patients with PAS, and investigated their roles in the overall survival (OS) of PAS.

## Materials and methods

### Patients

From May 2017 to March 2021, eligible and treatment-naïve patients with suspicious malignancies in the pulmonary artery from a consecutive population who underwent ^18^F-FDG PET/CT examination in two hospitals were retrospectively included in this study. The clinical data of the enrolled patients were collected, including age, gender, clinical symptoms (dyspnea, chest tightness, chest pain, cough, hemoptysis, and fever), blood D-dimer level, and lesion location.

### ^18^F-FDG PET/CT scan

PET/CT scans were performed using a Biograph 16 HR (Siemens Healthineers, Erlangen, Germany) or a Biograph 64 system (Siemens Healthineers, Erlangen, Germany). All patients were inquired to fast and avoid strenuous exercise at least 6 h before ^18^F-FDG injection, and the level of fasting blood glucose was no more than 7.0 mmol/L. Images were acquired approximately 60 ± 5 min after intravenous injection of 3.7 MBq of ^18^F-FDG per kilogram of body weight. Six or seven-bed positions were imaged from the base of the skull to the mid-thigh. PET images were obtained for 2–3 min per bed position. All image reconstructions were performed with the ordered-subset expectation maximization algorithm, incorporating a CT-based transmission map. PET images were reconstructed at 200 × 200 pixels using a Gaussian filter of 5.0 mm full width at half maximum value.

### PET/CT imaging analysis

PET metabolic parameters were analyzed on syngo station (Siemens Healthineers, Erlangen, Germany). Region of interest (ROI) was manually drawn on the primary lesion of the pulmonary artery, and the maximum value of an ROI was defined as the maximum standard uptake value (SUVmax). Metabolic tumor volume (MTV) of the lesion was computed with 40% of SUVmax as threshold, and total lesion glycolysis (TLG) of the lesion was calculated according to the following formula: TLG = SUVmean × MTV. PET/CT imaging results were analyzed and interpreted by two experienced nuclear medicine physicians who were unaware of the patients’ clinical information, other imaging, and pathology results. In cases of discrepancy regarding PET/CT findings, a consensus was reached after mutual discussion between them.

### Computed tomography pulmonary angiography (CTPA)

CTPA examinations were performed using a scanner of Brilliance iCT (Royal Philips, Amsterdam, Netherlands) or Revolution CT (GE Healthcare, Milwaukee, WI, USA), with the intravenous administration of iodinated contrast agent (iopamidol, 370 mg I/ml), which was modulated according to the patient weight. Non-enhanced and enhanced CT attenuation values (Hounsfield units, HU) were measured and the enhancement degree was described as mild or severe when the CT value increased (ΔCT value) by < 20 HU or ≥ 20 HU, respectively.

### Follow-up

All enrolled patients underwent routine clinical follow-up, which included clinical symptoms, laboratory test results, imaging data, and histopathological findings. OS was defined as the time interval from PET/CT scan to the date of death of any cause or until the end of December 2021.

### Statistical analysis

SPSS 25.0 software (IBM Corp., Armonk, NY, USA) was used for statistical analysis in the current study. Pearson's Chi-square (χ2 test) or Fisher's exact tests were carried out to evaluate differences in distribution of categorical variables, while the independent t-test or Mann–Whitney U test was performed for continuous variables. Continuous variables were expressed as the median (range minimum–maximum). Receiver operating characteristic (ROC) curve analysis was performed to determine the ability of SUVmax in distinguishing PAS from PTE, and the cutoff value was identified using the Youden index. Kaplan–Meier survival analysis was performed to predict the OS. The predictive value of FDG PET/CT parameters and clinical factors was analyzed via univariate Cox proportional hazards regression. *P* < 0.05 was considered to be statistically significant.

## Results

### Clinical characteristics between PAS and PTE

A total of 14 patients with PAS and 33 patients with PTE were enrolled in this study, and the patients’ characteristics were listed in Table 1. Among 14 patients with PAS, there were 9 men and 5 women with a median age of 50 years (range 27–69 years). And 33 PTE patients consisted of 20 men and 13 women with a median age of 62 years (range 15–88 years). PAS and PTE mainly presented dyspnea, chest tightness, chest pain, cough, hemoptysis, and fever, of which dyspnea and cough were the most common. Blood D-dimer examinations were performed in 13 patients with PAS and in all patients with PTE, and the abnormal results were found in 9 PAS patients (69.2%) and 30 PTE patients (90.9%). Pulmonary trunk (PT) involvement was found in 78.6% (11/14) PAS patients and 9.1% (3/33) PTE patients. Compared with PTE, PAS more frequently occurred in younger age (*P* = 0.011) and involved with PT (*P* < 0.001). No significant differences in gender, clinical symptom, and D-dimer level were found between the two groups (*P* > 0.05).

**Table 1:** Clinical, radiological, and PET/CT characteristics of the enrolled patients

Characteristics	All patients	PAS	PTE	*P* value
Age (median, range, years)	59 (15–88)	50 (27–69)	62 (15–88)	0.053
< 59, n	23	11	12	0.011*
≥ 59, n	24	3	21	
Gender, n				
Male	29	9	20	0.812
Female	18	5	13	
Symptom, n				
Dyspnea				
Yes	19	8	11	0.128
No	28	6	22	
Chest tightness				
Yes	12	4	8	0.731
No	35	10	25	
Chest pain				
Yes	12	6	6	0.076
No	35	8	27	
Cough				
Yes	20	7	13	0.501
No	27	7	20	
Hemoptysis				
Yes	5	2	3	0.627
No	42	12	30	
Fever				
Yes	6	2	4	1.000
No	41	12	29	
D-dimer level, n				
Abnormal	39	9	30	0.087
Normal	7	4	3	
Location, n				
With PT involved	14	11	3	< 0.001*
PT only	2	1	1	
PT and RPA	3	3	0	
PT and LPA	1	1	0	
PT, RPA, and LPA	8	6	2	
Without PT involved	33	3	30	
RPA	16	2	14	
LPA	6	1	5	
RPA and LPA	11	0	11	
Non-enhanced CT value (HU)	36 (18–63)	34 (18–47)	39 (24–63)	0.062
< 36, n	21	8	13	0.263
≥ 36, n	26	6	20	
Enhanced CT value (HU)	49 (26–76)	67 (31–76)	45 (26–64)	< 0.001*
< 49, n	23	3	20	0.024*
≥ 49, n	24	11	13	
ΔCT value (HU)	5 (0–44)	25 (13–44)	4 (0–18)	< 0.001*
< 20, n	36	3	33	< 0.001*
≥ 20, n	11	11	0	
SUV_max_	2.0 (0.8–17.2)	8.9 (3.0–17.2)	1.8 (0.8–3.7)	< 0.001*
< 2.0, n	21	0	21	< 0.001*
≥ 2.0, n	36	14	12	

*PAS* pulmonary artery sarcoma, *PTE* pulmonary thromboembolism, *PT* pulmonary trunk, *RPA* right pulmonary artery, *LPA* left pulmonary artery

*Indicated statistically significant

### ^18^F-FDG PET/CT and CTPA between PAS and PTE

**Fig. 1:**
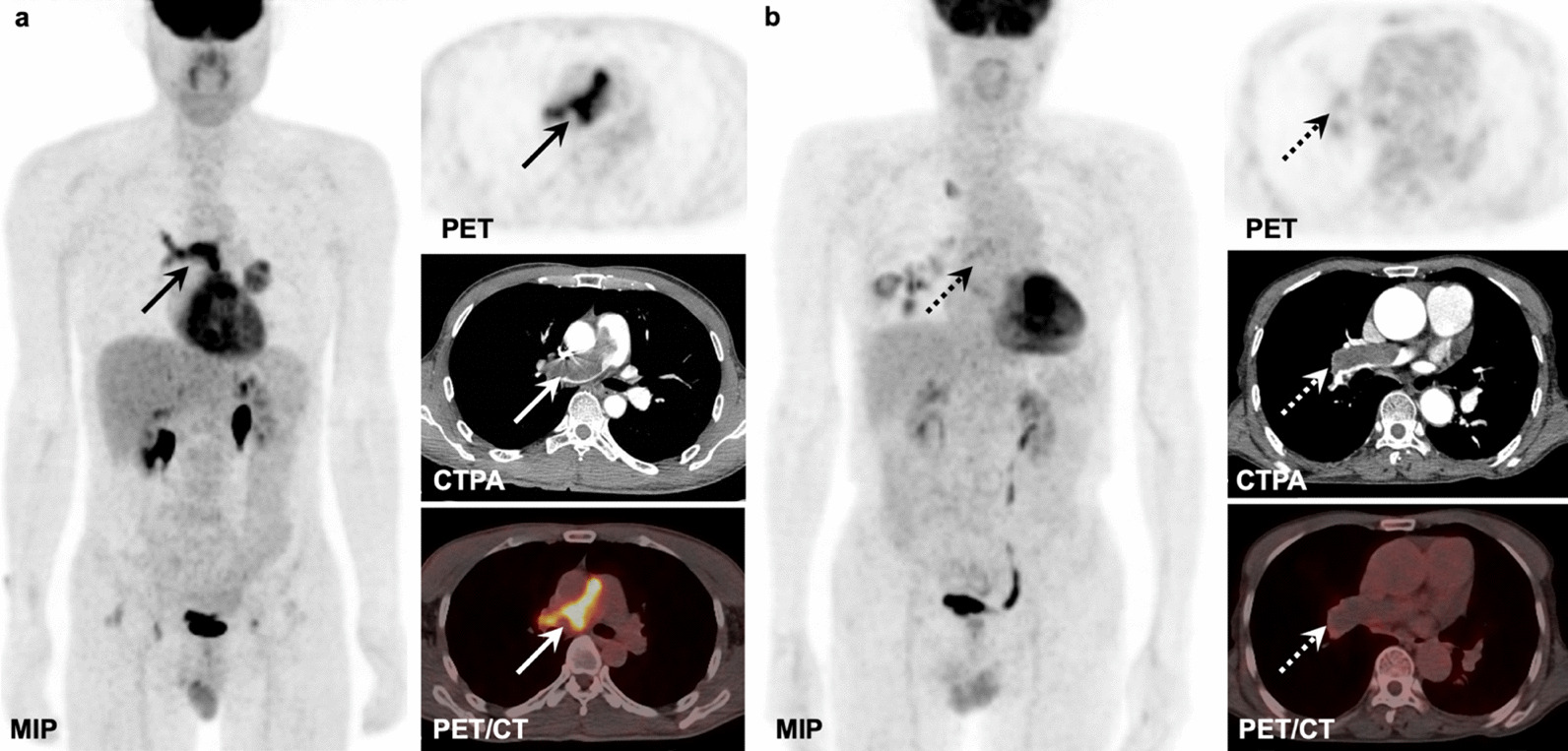
**a** PAS in a 52-year-old man involving PT and RPA (solid arrows) with intense FDG uptake (SUVmax: 10.7) on the MIP, axial PET, and fused PET/CT images, and filling defect on the CTPA image. **b** PTE in a 64-year-old man involving RPA (dotted arrows) with mild FDG uptake (SUVmax: 1.1) on the MIP, axial PET, and fused PET/CT images, and filling defect on the CTPA image

**Fig. 2:**
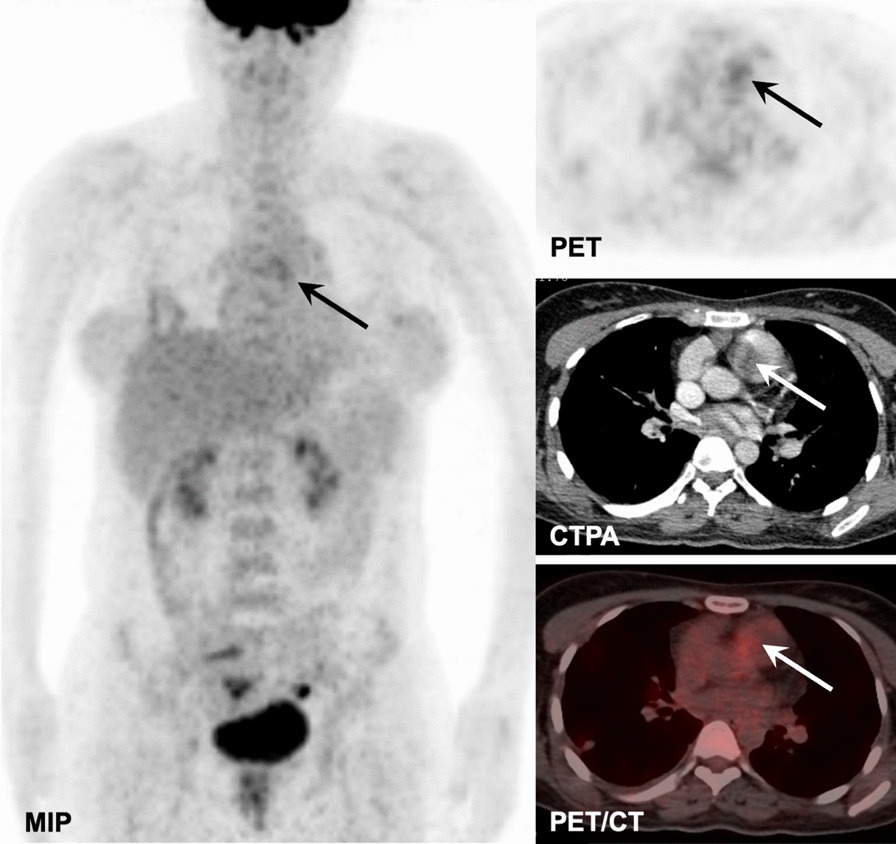
PAS in a 37-year-old woman involving PT and RPA (solid arrows) with moderate FDG uptake (SUVmax: 3.0) on the MIP, axial PET, and fused PET/CT images, and filling defect on the CTPA image

**Fig. 3:**
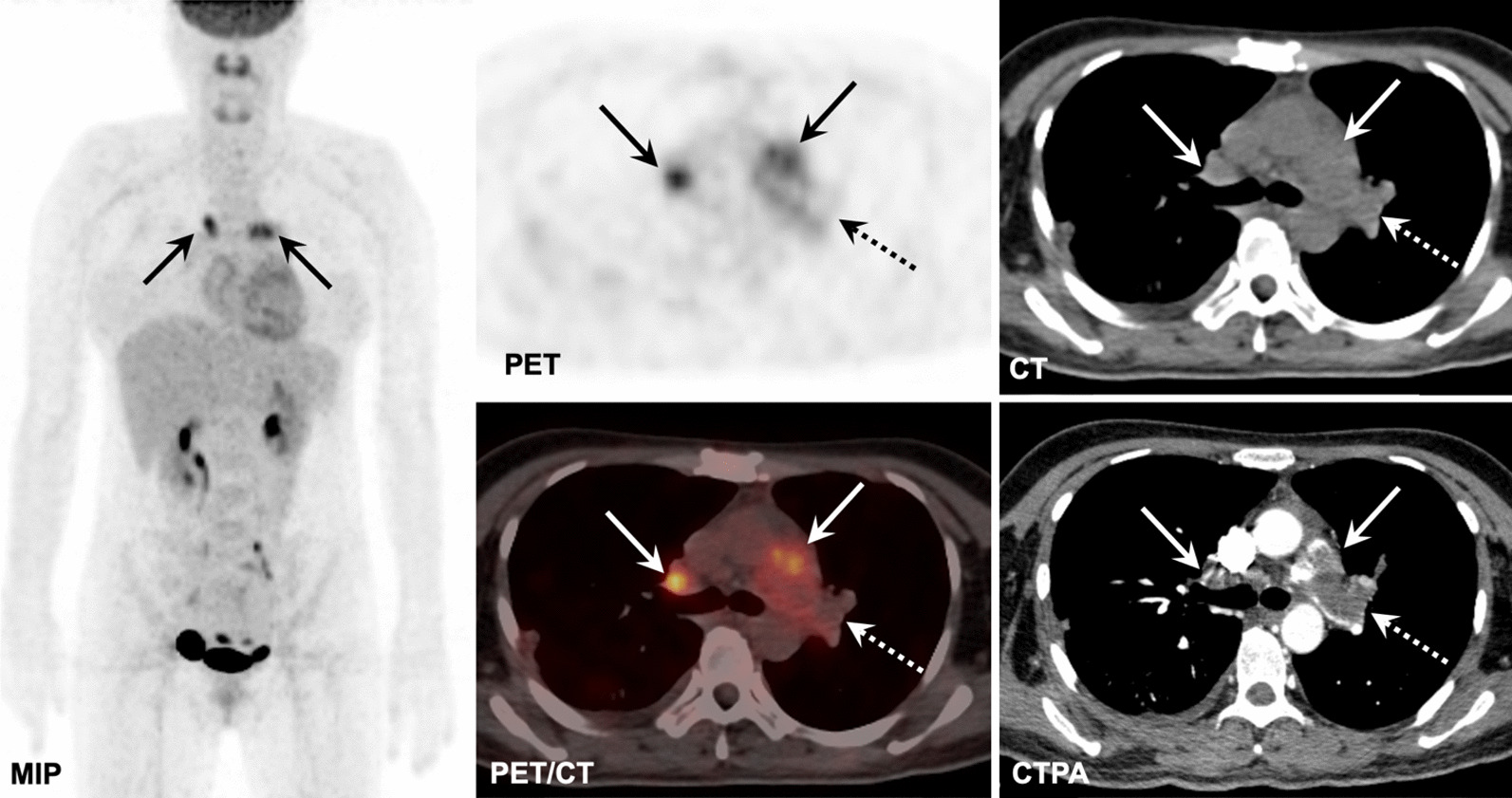
A 44-year-old woman detected with the coexistence of PAS involving PT, RPA, and LPA with SUVmax of 6.2 (solid arrows), and PTE involving LPA with SUVmax of 1.6 (dotted arrows) on FDG PET/CT and CTPA images

**Fig. 4:**
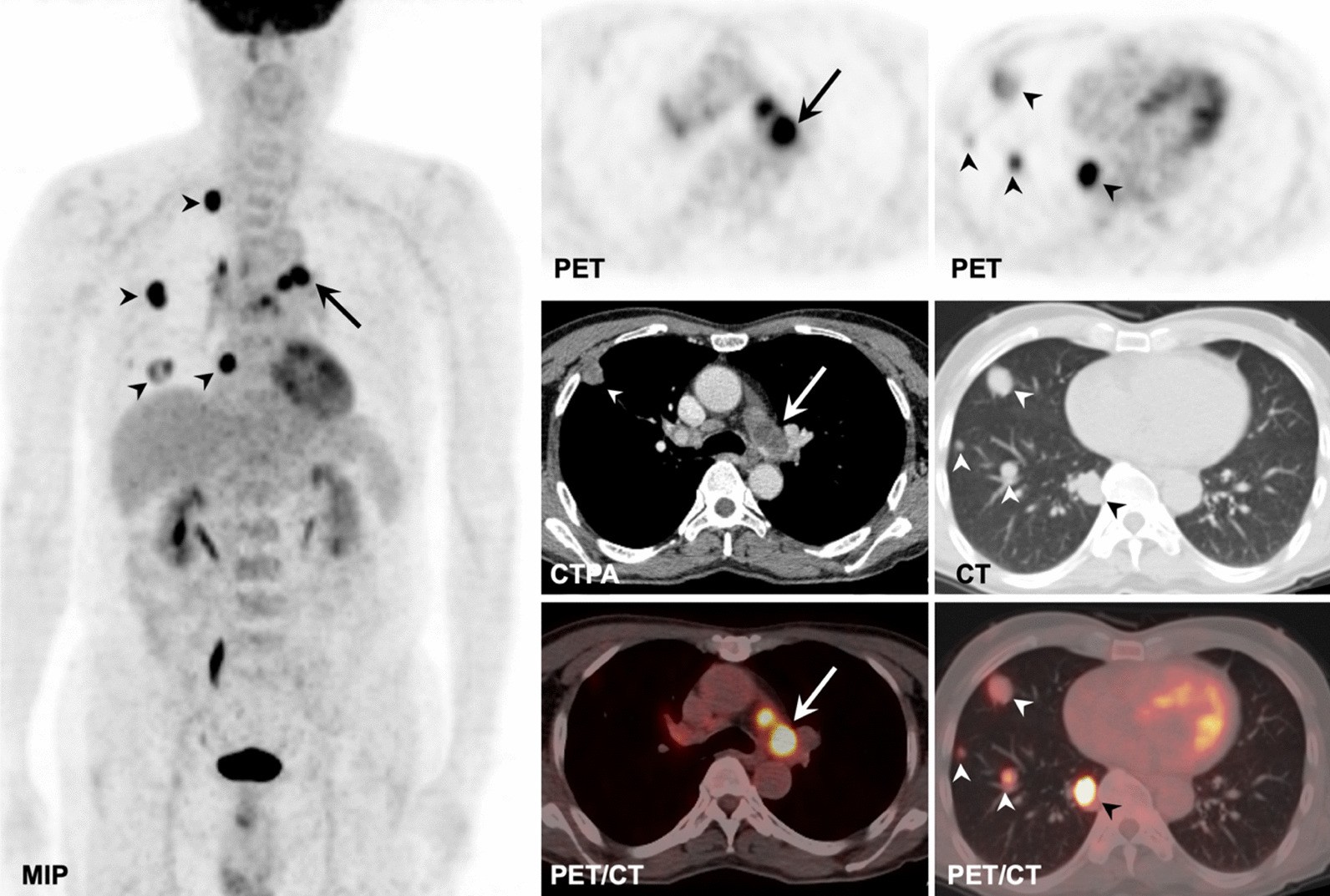
PAS in a 67-year-old man involving PT, RPA, and LPA (solid arrows) with SUVmax of 11.3 on the MIP, axial PET, and fused PET/CT images and filling defect on the CTPA image, with the metastases of right lung (arrowheads) on the MIP, axial PET, CT, and fused images and mediastinal and right hilar lymph nodes

**Fig. 5:**
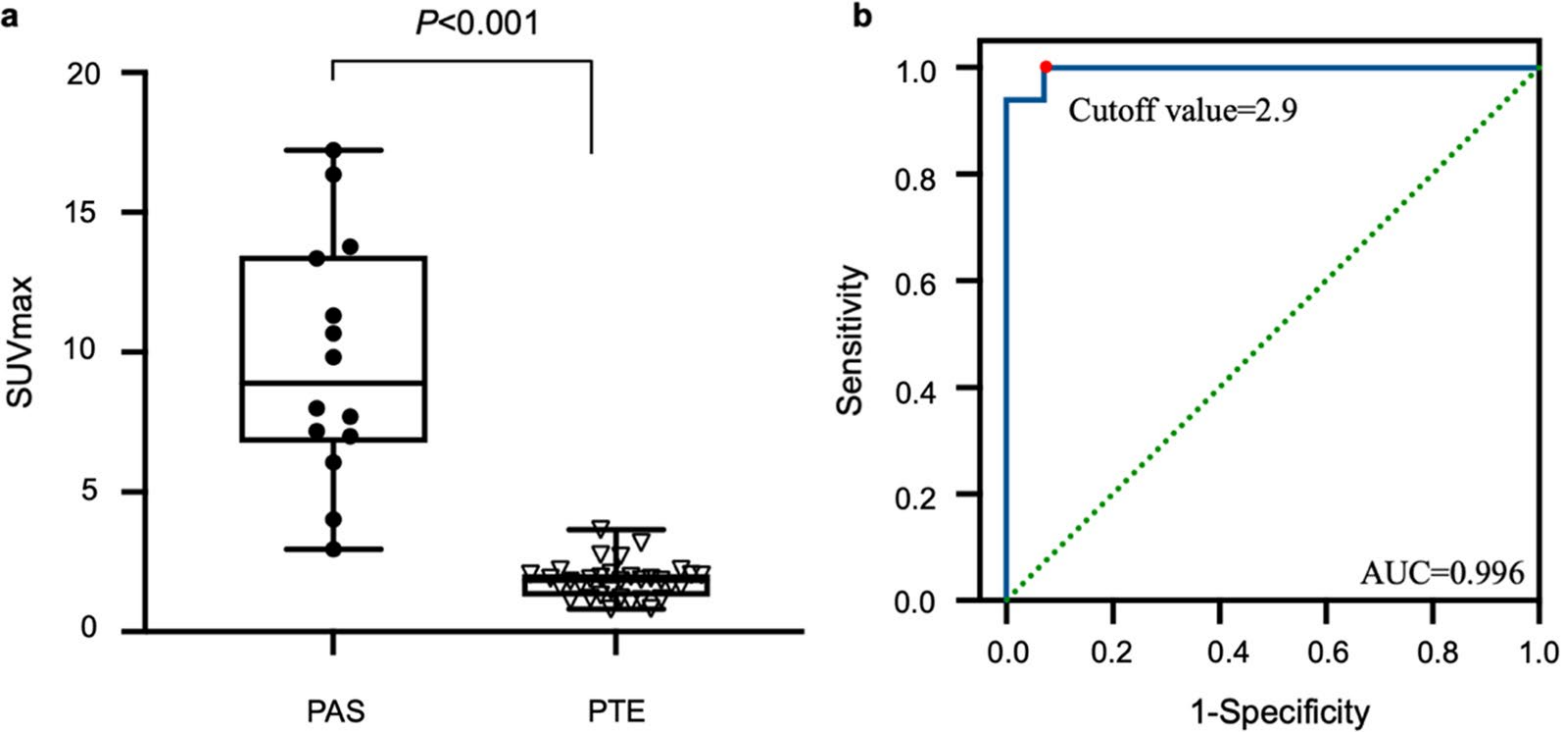
(a) The difference in SUVmax between PAS and PTE groups (*P* < 0.001). (b) Using ROC to identify the best cutoff value of SUVmax for distinguishing PAS from PTE

The median SUVmax of PAS and PTE was 8.9 (range 3.0–17.2) and 1.8 (range 0.8–3.7), respectively (Figs. 1, 2, 3, 4). The SUVmax of PAS was significantly higher than that of PTE (*P* < 0.001, Fig. 5a). Based on the ROC analysis, the best cutoff value of SUVmax for the differential diagnosis of PAS and PTE was 2.9, the area under the curve (AUC) was 0.996 (95% CI = 0.984–1.000) (Fig. 5b), and the sensitivity and specificity were 100.0% and 93.9%, respectively.

**Fig. 6:**
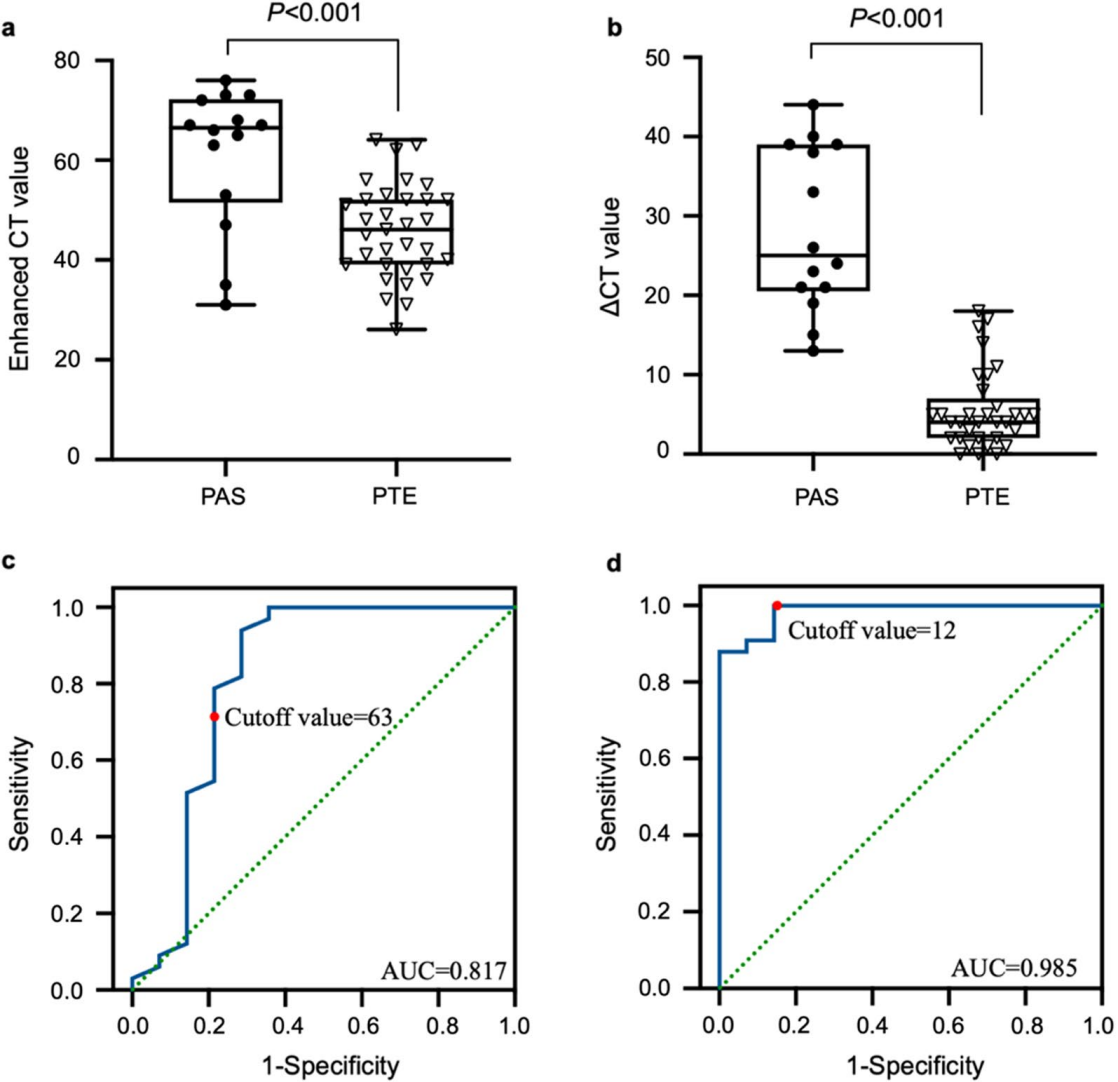
The differences in enhanced CT (**a**) and ΔCT (**b**) values between PAS and PTE groups (*P* < 0.001). Using ROC to identify the best cutoff value of enhanced CT (**c**) and ΔCT (**d**) for distinguishing PAS from PTE, respectively

The median non-enhanced CT values in PAS and PTE groups were 34 HU (range 18–47 HU) and 39 HU (range 24–63 HU), respectively, and no significant difference was noted (*P* > 0.05). Among all 47 cases, 36 cases displayed mild enhancement with ΔCT < 20 HU, and 11 cases showed severe enhancement with ΔCT ≥ 20 HU. The median enhanced CT values in PAS and PTE groups were 67 HU (rang, 31–76 HU) and 45 HU (range 26–64 HU), respectively, and the median ΔCT values were 25 HU (range 13–44 HU) and 4 (range 0–18 HU), respectively. There were significant differences in enhanced CT (*P* < 0.001, Fig. 6a) and ΔCT (*P* < 0.001, Fig. 6b) values between the two groups. Based on the ROC analysis, the best cutoff values of enhanced CT and ΔCT for the differential diagnosis of PAS and PTE were 63 HU and 12 HU, respectively, and the area under the curve (AUC) was 0.817 (95% CI = 0.643–0.992) (Fig. 6c) and 0.985 (95%CI = 0.959–1.000) (Fig. 6d), respectively. The sensitivity and specificity of enhanced CT were 71.4% and 93.9%, respectively, and the sensitivity and specificity of ΔCT were 100% and 87.9%, respectively.

### Correlation between PET/CT parameters and clinical and CT data in PAS

**Fig. 7:**
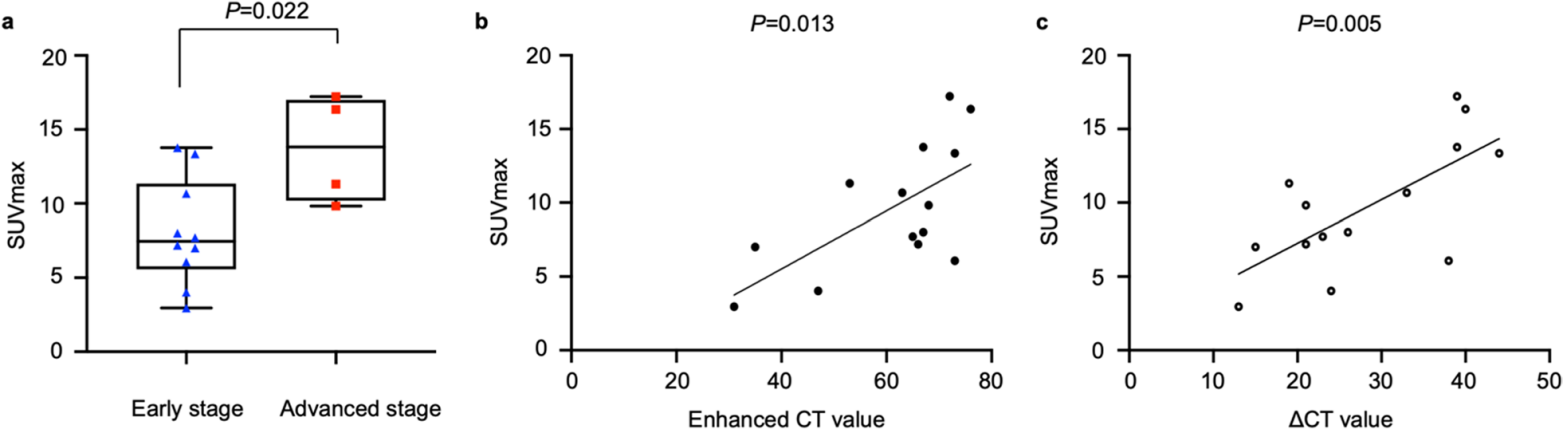
The difference in SUVmax between early and advanced stages of PAS (**a**, *P* = 0.022). The correlation of SUVmax with enhanced CT (**b**, r = 0.645, *P* = 0.013) or ΔCT (**d**, r = 0.702, *P* = 0.005) values

Detailed characteristics of the 14 patients with PAS were summarized in Table 2. The median SUVmax of early and advanced stages was 7.5 (range 3.0–13.8) and 13.8 (range 9.8–17.2), respectively, and significant difference was observed (*P* = 0.022, Fig. 7a). SUVmax was positively related to enhanced CT (r = 0.645, *P* = 0.013, Fig. 7b) and ΔCT (r = 0.702, *P* = 0.005, Fig. 7c) values. Moreover, SUVmax had no association with age, gender, symptom, D-dimer level, lesion location, and non-enhanced CT value (*P* > 0.05).

**Table 2:** Detailed characteristics of the 14 patients with PAS

Patient no.	Age (years)	Gender	D-dimer level	Tumor location	Tumor staging	Enhanced CT value (HU)	ΔCT value (HU)	SUVmax	MTV	TLG	OS (months)
1	57	M	N	LPA	II	66	21	7.2	26.8	114.2	3.0
2	33	F	A	RPA	I	67	26	8.0	12.1	55.0	21.0
3	69	F	N	PT, RPA, and LPA	III	76	40	16.4	7.6	62.9	0.2
4	57	M	N	PT, RPA, and LPA	III	68	21	9.8	–	–	1.0
5	40	M	A	PT	I	65	23	7.7	14.4	57.2	16.0
6	69	M	A	PT and RPA	II	67	39	13.8	11.2	90.8	10.0^#^
7	39	M	A	RPA	II	73	44	13.4	9.8	73.2	44.0
8	67	M	A	PT, RPA, and LPA	IV	53	19	11.3	7.5	50.0	censored
9	44	F	A	PT, RPA, and LPA	I	73	38	6.1	17.9	60.6	censored
10	52	M	A	PT, RPA, and LPA	II	63	33	10.7	23.7	142.0	11.0^#^
11	27	F	A	PT and LPA	IV	72	39	17.2	10.7	114.8	5.0
12	37	F	N	PT and RPA	I	31	13	3.0	5.4	10.7	15.0
13	47	M	-	PT and RPA	II	47	24	4.0	14.9	35.1	14.0
14	56	M	A	PT, RPA, and LPA	II	35	15	7.0	21.8	81.0	2.0

*M* male, *F* female, *N* normal, *A* abnormal, *PT* pulmonary trunk, *RPA* right pulmonary artery, *LPA* left pulmonary artery, *OS* overall survival

^#^To the end of December 2021

Except for the measurement of metabolic volume in one patient influenced by the physiological FDG uptake of heart, the median MTV and TLG of the remaining 13 patients were 12.1 (range 5.4–26.8) and 62.9 (range 10.7–142.0), respectively. No significant association was found between MTV (or TLG) and clinical or CT findings (*P* > 0.05).

### Follow-up of PAS

During the follow-up period, 2 patients were lost to follow-up. Among the rest 12 patients, surgical treatment was performed in 5 patients, non-surgical treatment was performed in 5 patients, including chemotherapy, radiotherapy, and immunotherapy, and no treatment was in 2 patients. Disease progression was found in 11 out of 12 patients including 10 deaths, and stable condition was showed in one case. All 10 deaths were not owing to surgery but disease-related.

The follow-up duration was 11.8 ± 12.2 months, ranging from 0.2 to 44.0 months.

**Fig. 8:**
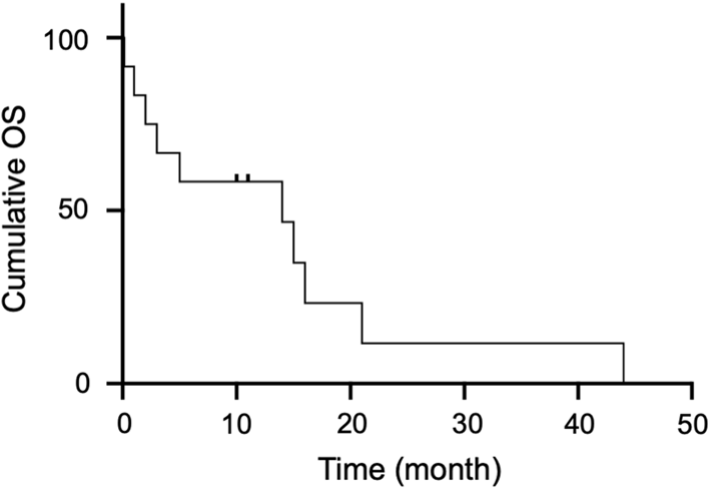
The Kaplan–Meier curve of OS in patients with PAS

Univariate Cox regression analysis showed that only D-dimer level (*P* = 0.038) and tumor staging (*P* = 0.019) were significantly associated with OS (*P* < 0.05, Table 3). Kaplan–Meier survival analysis presented that the median OS was 10.5 months, and 12-month and 24-month OS rates were 58.0% and 12.0%, respectively (Fig. 8).

**Table 3:** Univariate Cox proportional hazards regression for OS in the patients with PAS

Variables	Univariate analysis		
	*P* value	HR	95% CI
Age	0.079	7.283	0.797–66.522
Gender	0.650	0.735	0.194–2.783
D-dimer	0.038*	0.161	0.029–0.906
Location	0.164	4.611	0.536–39.678
Tumor staging	0.019*	8.933	1.444–55.275
Non-enhanced CT value	0.246	2.291	0.564–9.298
Enhanced CT value	0.538	0.634	0.149–2.705
ΔCT value	0.088	0.158	0.019–1.312
SUV_max_	0.686	0.746	0.181–3.075
MTV	0.340	2.093	0.459–9.544
TLG	0.719	0.761	0.172–3.371

*Indicated statistically significant

*HR* hazard ratio, *CI* confidence interval

## Discussion

The current study showed that PAS patients were significantly younger than PTE patients, and there was no sex discrimination, which was similar to the literatures reported previously [10, 11]. PAS and PTE presented similar symptoms in our and previous studies [10, 12], including dyspnea, chest tightness, chest pain, cough, hemoptysis, fever, and so on, thus it was very difficult to distinguish PAS from PTE based on clinical symptoms. Besides, Guo et al. reported that D-dimer was within the normal range in 9 patients with PAS [13]. Li et al. found that D-dimer was elevated in 16 of 21 patients with PAS [12]. Our study showed that abnormal D-dimer levels were existed in 9 of 13 PAS patients and 30 of 33 PTE patients, which demonstrated that the routine laboratory test of D-dimer didn’t show good diagnostic efficacy. Lastly, although PT and right pulmonary artery (RPA) were much more commonly involved by PAS, as previously reported [12], we found that PAS more frequently involved PT. Thus, pulmonary artery malignancies should be listed in the differential diagnosis in the case of lesion invasion into PT.

CTPA is the most common imaging modality for patients with pulmonary artery diseases. Filling defects within the pulmonary artery are easily detected on CTPA. However, due to the rare and fatal nature of PAS, some clinicians and radiologists don’t know enough about it, which is easily misdiagnosed as PTE. Ito et al. reported [14] that there was no significant difference in enhanced CT value between PAS and PTE. Conversely, significant differences were observed in the enhanced CT and ΔCT values between the two groups in this study. We assume that CT enhancement can reflect the hemodynamic characteristics of lesions, which is used to evaluate the malignancy and invasion of tumors [15, 16].

18F-FDG PET/CT has been reported to be useful in distinguishing PAS from PTE based on SUVmax. Ito et al. reported that the SUVmax of 3 PAS cases (7.6 ± 2.2) was significantly higher than that of 10 PTE cases (2.3 ± 0.4) [14]. Xi et al. reported the obvious difference in SUVmax observed between 11 PAS patients (11.1 ± 4.9) and 9 PTE patients (2.3 ± 0.4) [17]. Our study also displayed that the SUVmax of 14 PAS patients was significantly higher than that of 33 PTE patients. Moreover, Li et al. demonstrated that 13 out of 14 PAS cases had increased FDG uptake ranging from 2.8 to 15.4, and one case was regarded as FDG-negative [12]. Rosales Castillo et al. also illustrated that one PAS case with low FDG uptake was misdiagnosed as PTE [18]. For the current study, one case with PAS also displayed relatively low FDG uptake and the SUVmax was 3.0. Poor or low FDG uptake in PAS was possibly attributable to low malignant cellularity and abundant thrombus and fibrous tissue [19, 20]. Moreover, increased FDG uptake in PTE was also reported [21]. The thrombus consisted of organized tissue and inflammatory cells, and high FDG uptake was possibly related to the numbers of macrophages or neutrophils [22, 23]. Therefore, the overlap of glucometabolism in the two groups should be kept in mind in the clinical practice.

At present, the sample size of the studies about PET/CT in PAS was quite limited, mostly less than 15 patients [11]. There is still no report about the association between ^18^F-FDG PET/CT metabolic parameters and clinical and radiological features in PAS. For this study, SUVmax, MTV, and TLG of PAS were investigated with age, gender, D-dimer level, tumor location, staging, and CT values, and the results showed that only SUVmax was associated with tumor staging and enhanced CT and ΔCT values. Furthermore, combined the SUV > 2.9 with ΔCT > 12 HU in this study, the sensitivity and specificity of diagnosing PAS can reach 100%.

Surgical resection is the most common therapy for PAS, but the prognosis is still very poor [24]. Our study showed that the median OS time was 10.5 months, similar to the study of Li et al. [12]. Univariate Cox analysis demonstrated that only D-dimer level and tumor staging were associated with OS. However, multivariate Cox analysis was not further analyzed owing to the small sample. It is proposed that timely diagnosis as well as complete surgical resection can improve the prognosis of the disease [12, 25]. Despite the fact that PET/CT metabolic parameters were not associated with OS in this study, the utility of ^18^F-FDG PET/CT can effectively reduce a delay in diagnosis, accurately detect the involved extent of tumor, and quickly assist clinicians to select the suitable treatment, which is helpful to improve the prognosis.

There are also some limitations to this study. Firstly, the sample size was small, and further large-scale studies of ^18^F-FDG PET/CT in this kind of rare tumor are warranted. Secondly, there are many pathological subtypes of PAS, including intimal sarcoma, leiomyosarcoma, undifferentiated pleomorphic sarcoma, and others that could not be subtyped. The role of FDG PET/CT in different pathological subtypes of PAS should be further investigated. Finally, tumor embolism is also the common disease of pulmonary artery. The comparison of FDG PET/CT among PAS, PTE, and tumor embolism should be explored in future study.

## Conclusions

Most PAS displayed high glucose metabolism, and SUVmax of ^18^F-FDG PET/CT was a useful imaging modality for distinguishing PAS from PTE. SUVmax was associated with tumor staging and enhanced CT and ΔCT values. The role of FDG PET/CT in predicting the prognosis of PAS should be explored in the large-scale studies.

## Data Availability

The datasets used and/or analyzed during the current study are available from the corresponding author on reasonable request.

## Abbreviations

PAS: Pulmonary artery sarcoma
PTE: Pulmonary thromboembolism
OS: Overall survival
SUVmax: Maximum standard uptake value
MTV: Metabolic tumor volume
TLG: Total lesion glycolysis
CTPA: Computed tomography pulmonary angiography
HU: Hounsfield units
ROC: Receiver operating characteristic
AUC: Area under the curve
PT: Pulmonary trunk
RPA: Right pulmonary artery
LPA: Left pulmonary artery
